# Mapping the ChOLE classification to hearing outcomes and disease-specific health-related quality of life

**DOI:** 10.1007/s00405-020-06002-x

**Published:** 2020-05-05

**Authors:** Nora M. Weiss, David Bächinger, Adrian Rrahmani, Hans E. Bernd, Alexander Huber, Robert Mlynski, Christof Röösli

**Affiliations:** 1 Department of Oto-Rhino-Laryngology, Head and Neck Surgery “Otto Körner”, Rostock University Medical Center, Doberaner Strasse 137-139, 18057 Rostock, Germany; 2 Department of Otorhinolaryngology, Head and Neck Surgery, University Hospital Zurich, Zurich, Switzerland; 3 University of Zurich, Zurich, Switzerland

**Keywords:** Tympanoplasty, Cholesteatoma, Quality of life, ChOLE classification, Staging, ZCMEI-21

## Abstract

**Objectives:**

To investigate the association between the “ChOLE” classification, hearing outcomes and disease-specific health-related quality of life (HRQoL).

**Methods:**

In two tertiary referral centers, patients requiring primary or revision surgery for cholesteatoma were assessed for eligibility. Audiometric assessment was performed pre- and postoperatively. The ChOLE classification was determined intraoperatively and via the preoperative CT scan. HRQoL was assessed pre- and postoperatively using the Zurich Chronic Middle Ear Inventory (ZCMEI-21).

**Results:**

A total of 87 patients (mean age 45.2 years, SD 16.2) were included in this study. ChOLE stage I cholesteatoma was found in 8 (9%), stage II cholesteatoma was found in 65 (75%), and stage III cholesteatoma was found in 14 (16%) patients. Postoperatively, the mean air–bone gap (0.5, 1, 2, 3 kHz) was significantly smaller than before surgery (14.3 dB vs. 23.0 dB; *p* = 0.0007). The mean ZCMEI-21 total score significantly decreased after surgery (26.8 vs. 20.7, *p* = 0.004). No correlation between the ZCMEI-21 total score and both the ChOLE stage and the extent of the cholesteatoma (ChOLE subdivision “Ch”) was found. A trend towards worse HRQoL associated with a poorer status of the ossicular chain (ChOLE subdivision “O”) was observed. The audiometric outcomes were not associated with the extent of the cholesteatoma. The ChOLE subdivision describing the ossicular status showed a strong association with the pre- and postoperative air conduction (AC) thresholds. Further, the ZCMEI-21 total score and its hearing subscore correlated with the AC thresholds.

**Conclusion:**

The ChOLE classification does not show a clear association with HRQoL measured by the ZCMEI-21. The HRQoL neither seems to be associated with the extent of the disease nor with the ossicular chain status. Yet, surgical therapy significantly improved HRQoL by means of reduced ZCMEI-21 total scores, which were strongly associated with the AC thresholds. Intraoperative assessment of a cholesteatoma using the ChOLE classification and HRQoL complement each other and provide useful information.

## Introduction

Cholesteatoma is a progressive disease that may impair the patient’s quality of life [1] and bears the risk of severe complications such as meningitis [2]. Its diagnosis always leads to an indication for surgery with the principle of complete removal of the keratinizing epithelium from the mastoid and middle ear including hearing restoration [3]. Due to the frequent use of subjective descriptions by the surgeons, e.g., concerning the extent of the cholesteatoma, an objective comparison of the surgical techniques and outcomes is hindered. Classification systems may facilitate comparing outcomes and surgical techniques. To support uniform reporting systems, multiple classification systems have been suggested in the past [4–7]. Most commonly, the localization of the adhesion or the retraction pocket was involved and considered important [6, 8]. Cholesteatomas affecting the pars tensa were found to have a poorer hearing outcome after surgery [6, 9].

Current cholesteatoma classifications aim to facilitate the surgeons decision for the correct approach during surgery according to the extent of the cholesteatoma [4, 10]. Further, cholesteatoma classifications allow comparing surgical and audiometric outcomes between surgeon and centers. The surgical approach depends on the presence of complications such as abscess, labyrinthitis, facial palsy, or on the pneumatization and ventilation of the mastoid [11]. Additionally, the structures involved in the disease such as the ossicles need to be taken into account since they limit the outcome and the intraoperative risk [7, 12]. Former classifications such as from Sanna et al. [4], Rosito et al. [5], Black et al. [6] or from the European Academy of Otology and Neurotology (EAONO) and the Japanese Otological Society (JOS) [13] focus on growth patterns, extension and approaches for the extent of the cholesteatoma and lack information about the mastoid aeration, the status of the ossicular chain or clinical symptoms. To take these aspects into account, they gave reason for revision that also include these additional information [14]. Yet, a novel comprehensive classification system has been recently developed, i.e. the ChOLE classification [15]. The ChOLE classification has several advantages over existing classification. The advantages include the possibility of classifying apical or supra/infralabyrinthine cholesteatomas, as well as the inclusion of the degree of mastoid pneumatization and ventilation [15]. The ChOLE classification consists of four subdivisions: first, the extent of the cholesteatoma inside the middle ear and temporal bone (Ch); second, the status of the ossicular chain (O); third, life-threatening complications (L) such as facial palsy, labyrinthitis or meningitis; and last, the Eustachian tube function as defined by the aeriation of the temporal bone (E).

Even though two recent studies investigated the association of the cholesteatoma extent and hearing outcomes [16, 17], only few studies evaluated the impact of cholesteatoma surgery on health-related quality of life (HRQoL) [1, 18–20]. To our knowledge, no studies exist investigating the association between cholesteatoma stages as defined by a classification system and HRQoL. Thus, the aim of this study was to evaluate the new ChOLE classification in a clinical setting and to map it to HRQoL as measured by the Zurich Chronic Middle Ear Inventory (ZCMEI-21).

## Materials and methods

### Ethical consideration

The study protocol was approved by the local Ethics Committees in accordance with the Helsinki declaration (registration-number: A2017-0101 Rostock, Germany; No. 2018–02216, Zurich, Switzerland). Informed consent was obtained from all the participants.

### Patient selection

Patients receiving middle ear surgery between April 2016 and October 2019 due to primary or recurrent cholesteatoma from two tertiary referral centers in Switzerland and Germany were assessed for inclusion into the study. To evaluate the association of the ChOLE classification and the subjective benefit from surgery, only patients who completed the ZCMEI-21 postoperatively were included into analyses covering postoperative outcomes. All patients received a CT scan prior surgery to assess the middle ear anatomy and the aeriation of the mastoid. Each patient was asked to complete the ZCMEI-21 before and after surgery.

### Audiometric assessment

All audiometric measurements were performed with calibrated instruments in a sound-proof room (DIN EN ISO 8253) by audiological trained stuff. The air–bone gap (ABG) was calculated as the difference between the pure-tone average (PTA) of the air conduction (AC) and bone conduction (BC) thresholds at 0.5, 1, 2, and 3 kHz (PTA_0.5–3 kHz_). According to recommendations for hearing reporting standards [21] and from the Committee on Hearing Equilibrium guidelines [22], the ABG_0.5–3 kHz_ (hereinafter referred to as ABG) was chosen for evaluating the results of treating conductive hearing loss. Audiometric assessment was performed pre- and postoperatively.

### Radiological assessment

Low-dose high-resolution or cone beam CT imaging without intravenous contrast enhancement of the temporal bones was performed as a routine preoperative investigation. Data were reconstructed separately for each temporal bone in the axial plane using a standard bone algorithm.

### Assessment of health-related quality of life

The ZCMEI-21 was used to assess HRQoL [23]. The ZCMEI-21 as a disease-specific questionnaire for chronic middle ear disease has been translated in several languages [24–26] and is used in clinical settings for research and clinical practice [23, 27].

The ZCMEI-21 consists of four subscales concerning ear signs and symptoms, hearing function, psychosocial impact and the use of medical resources. Answers are presented using a five-point Likert scale. High scores correlate with a poorer quality of life [23] and the minimal clinical important difference (MCID) is estimated to 5 [28]. The ZCMEI-21 was designed as a disease-specific instrument to assess HRQoL of life in patients suffering from chronic middle ear disease and may also be used after surgical interventions. The ZCMEI-21 was completed prior surgery and at the follow-up visit after surgery.

### Cholesteatoma classification

Surgical classification of cholesteatoma was performed using the ChOLE classification consisting of four subdivisions [15]. Cholesteatomas are classified by (1) extension with subdivisions Ch1 describing limited extension within the middle ear to Ch4 describing a petrous apex cholesteatoma, (2) status of the ossicular chain at the end of surgery with O0 indicating an intact ossicular chain to O4 indicating a fixed stapes only, (3) complications with L2 describing extracranial and L4 describing intracranial complications, and (4) Eustachian tube function as determined by the degree of mastoid pneumatization and ventilation with E0 indicating a good to E2 indicating a poor pneumatization and ventilation. Cholesteatoma staging (I–III) follows a numeric rule and can be performed using a freely available online software tool [29]. The ChOLE classification was assessed using intraoperative findings (subdivisons Ch, O and L) and preoperative CT imaging (subdivision E).

### Statistical analysis

All statistical tests were selected before data collection. Statistical analyses were performed using Prism (version 8, GraphPad Software, La Jolla, CA, USA). The significance level was set to *p* < 0.05. The assumption of normality was tested graphically using quantile–quantile plots. If not otherwise specified, data are presented as mean with standard deviation (SD) or absolute numbers with percentages. To compare means of > 2 groups, a one-way ANOVA and Tukey’s test as a post hoc multiple comparison procedure were used. A Chi-squared test was used to compare the ChOLE classification to three different groups of hearing outcome (deterioration: ABG shift > 5 dB; no change: ABG shift −5 dB to 5 dB; improvement: ABG shift < 5 dB).

### Results

A total of 87 patients (45 [52%] males and 42 [48%] females) with a mean age of 45.2 years (SD 16.2 years) were included in the study. The affected side was left in 47 (54%) cases and right in 40 (46%) cases. The mean follow-up period was 204 days (SD 173 days). Postoperative data were available from 62 patients of which 8 had missing questionnaire data, leaving 54 patients with data for analyses concerning postoperative ZCMEI-21 scores. Consequently, data from 87 patients were available for all preoperative analyses, data from 62 patients for postoperative hearing analyses and 54 patients for postoperative HRQoL analyses.

### Audiometric outcomes

The mean preoperative AC PTA was 47.1 dB HL (SD 22.7 dB) and the mean preoperative BC PTA was 24.1 dB HL (SD 21.0 dB). The mean preoperative ABG was 23.0 dB (SD 11.7 dB). Postoperatively, the mean AC PTA and BC PTA were 43.7 dB HL (SD 28.6 dB), and 27.9 dB HL (SD 28.7 dB), respectively. There was no statistically significant difference between the pre- and postoperative BC PTA (*p* = 0.68). The mean postoperative ABG was 14.3 dB (SD 10.8 dB), which was significantly decreased as compared to the preoperative ABG (*p* = 0.0007). Analyzing three different subgroups of hearing outcome (deterioration: ABG shift > 5 dB [*n* = 10; 16%]; no change: ABG shift −5 dB to 5 dB [*n* = 26; 42%]; improvement: ABG shift < 5 dB [*n* = 26; 42%]) no differences among the cholesteatoma stage (*p* = 0.29), extent (subdivision Ch; *p* = 0.24) and ossicular status (subdivision O; *p* = 0.20) were observed.

### ChOLE classification

The distribution of ChOLE stages (I–III) and the ChOLE subdivisions (Ch, O, L and E) is shown in Table 1.

**Table 1:** Distribution of ChOLE subdivisions and stages

	Ch *n* (%)	O *n* (%)	L *n* (%)	E *n* (%)	Stage *n* (%)
0	n. a	6 (7)	80 (92)	15 (17)	n. a
1/I	31 (36)	47 (54)	n. a	23 (26.5)	8 (9)
2/II	31 (36)	23 (26)	7 (8)	49 (56.5)	65 (75)
3/III	7 (8)	8 (10)	n. a	n. a	14 (16)
4	18 (20)	3 (3)	0 (0)	n. a	n. a
Total	87 (100)	87 (100)	87 (100)	87 (100)	87 (100)

### ZCMEI-21 scores

The mean preoperative ZCMEI-21 total score was 25.1 (SD 15.0) and the mean postoperative ZCMEI-21 total score was 20.7 (SD 13.2). The mean ZCMEI-21 total score shift was −6.1 (SD 14.9). This change was statistically significant (*p* = 0.004) and also corresponds to a clinically relevant improvement in HRQoL [28]. The mean preoperative ZCMEI-21 hearing subscore was 7.9 (SD 5.2) and the mean postoperative ZCMEI-21 hearing subscore was 6.9 (SD 4.6) describing a statistically significant ZCMEI-21 hearing subscore shift (*p* = 0.01).

### ChOLE classification and hearing

**Fig. 1:**
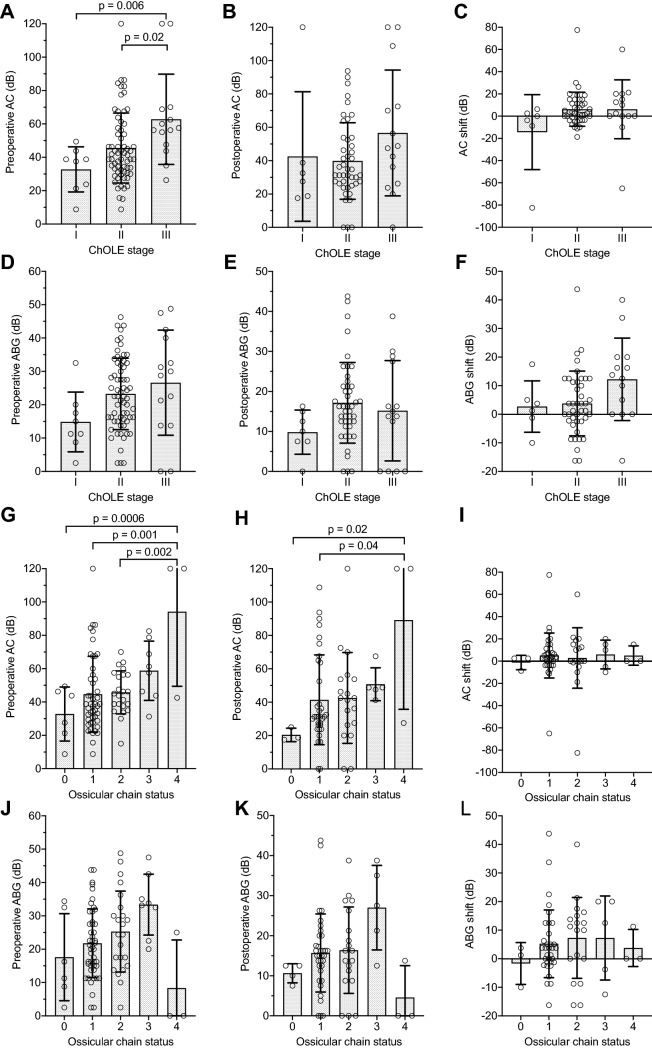
Association of audiometric outcomes and the ChOLE stage and its subdivision “O”. **a–c** Pre- and postoperative air conduction (AC) pure-tone average (PTA) compared to the ChOLE stage. **d–f** Pre- and postoperative ABG compared to the ChOLE stage. **g–i** Pre- and postoperative AC PTA compared to the ossicular chain status. **j–l** Pre- and postoperative ABG compared to the ChOLE subdivision “O” describing the status of the ossicular chain at the end of surgery. Bars represent mean, error bars indicate standard deviation

A significant association between the cholesteatoma stages and the preoperative AC PTA was found. A trend towards higher hearing thresholds for larger cholesteatomas was found with statistically significant differences between stage I and stage III cholesteatomas (*p* = 0.006) as well as between stage II and stage III cholesteatomas (*p* = 0.02; Fig. 1a). No such trend was observed postoperatively (*p* = 0.2, Fig. 1b) and further, no association between the ChOLE stage and neither the AC shift (Fig. 1c), the ABG (Fig. 1d–e) nor the ABG shift was found (Fig. 1f). No associations between the cholesteatoma extent (Ch) and any audiometric outcome were found. Concerning the ossicular chain status (O), an increased preoperative AC PTA with increasing subdivision “O” (Fig. 1g) was found; significant differences were observed between status O0 and O4 (*p* = 0.0006), O1 and O4 (*p* = 0.001), O2 and O4 (*p* = 0.002), but not between O3 and O4 (*p* = 0.09). Regarding postoperative AC PTA, the differences were only significant between subdivision O0 and O4 (*p* = 0.002) as well as between subdivision O1 and O4 (*p* = 0.004, Fig. 1h). No association was found between the subdivision “O” and the AC shift, the ABG, and the ABG shift (Fig. 1i–l).

### ChOLE classification and ZCMEI-21 scores.

**Fig. 2:**
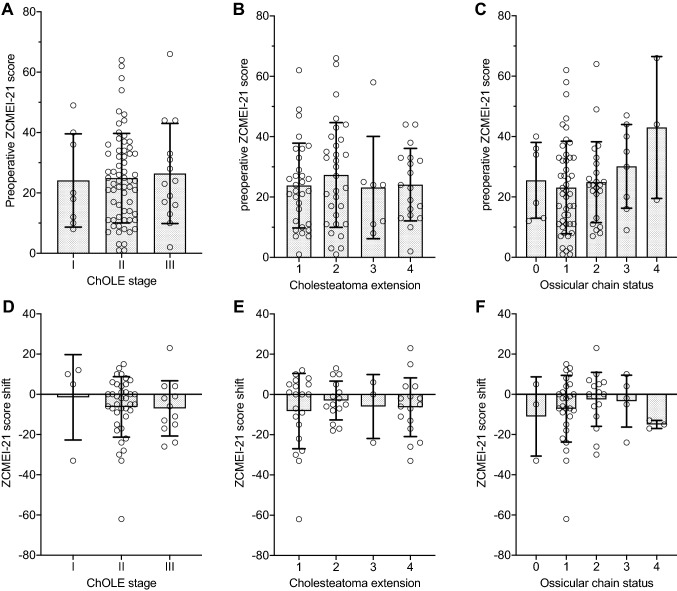
Association of the ChOLE stage and its subdivisions “Ch” and “O” with the ZCMEI-21 total score. **a–c** Association of preoperative ZCMEI-21 scores and the cholesteatoma stage (A), the cholesteatoma extension (B) and the ossicular chain status (C). **d–f** Association of ZCMEI-21 score shifts from pre- to postoperative values for the cholesteatoma stage (D), the cholesteatoma extension (E) and the ossicular chain status (F). A decrease in the ZCMEI-21 score indicates an improvement in HRQoL. Bars represent mean, error bars indicate the standard deviation. Bars represent mean, error bars indicate standard deviation

A total of 54 patients completed the ZCMEI-21 at the pre- and postoperative visit. No associations between the ChOLE classification and the ZCMEI-21 were observed for any of the tested hypotheses (Fig. 2). In particular, no association between the preoperative ZCMEI-21 total score and both the ChOLE stage (*p* = 0.92, Fig. 2a) and the subdivision “Ch” (*p* = 0.78, Fig. 2b) was found. Regarding the association between the preoperative ZCMEI-21 total score and the subdivision “O”, a trend towards a poorer ossicular chain status associated with higher ZCMEI-21 scores indicating a higher impairment in HRQoL (Fig. 2c) was found, although this association did not reach statistical significance (*p* = 0.20). Further, no association between the shift in the ZCMEI-21 total score and the ChOLE stage (*p* = 0.94, Fig. 2d), and the subdivisions “Ch” (*p* = 0.80, Fig. 2e) and “O” (*p* = 0.64, Fig. 2f) was found. As only seven patients (8%) exhibited an L subdivision greater than L0, no statistical analysis on the association between ZCMEI-21 and the L subdivision was performed.

### ZCMEI-21 score and hearing

**Fig. 3:**
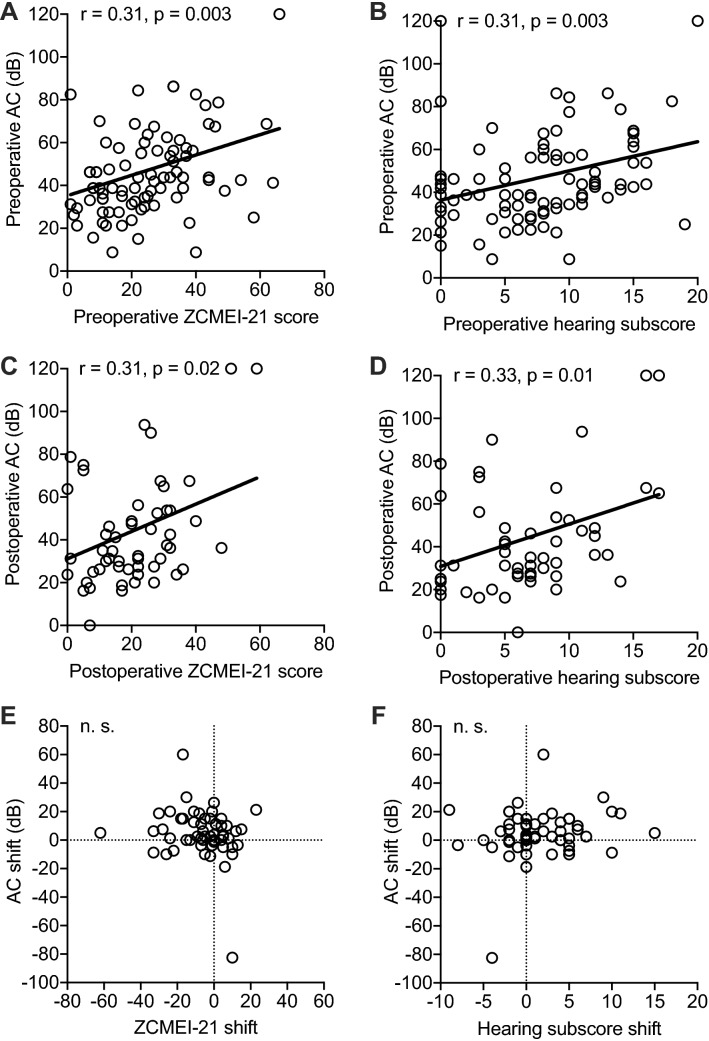
Association of the pre- and postoperative AC PTA with the ZCMEI-21. **a–b** Preoperative correlation between the AC PTA and the ZCMEI-21 total score (A) and the hearing subscore (B). **c–d** Postoperative correlation between the AC PTA and the ZCMEI-21 total score (C) and the hearing subscore (D). **e–f** Association between the AC shift and the ZCMEI-21 total score shift (E) and the hearing subscore shift (F). Solid line indicates linear regression line; *r*, Spearman’s rank correlation coefficient

A significant correlation between the AC threshold and the ZCMEI-21 total score preoperatively (*r* = 0.31; *p* = 0.003, Fig. 3a) and postoperatively (*r* = 0.31; *p* = 0.02, Fig. 3c) and also for the hearing subscore (ZCMEI-21 subscale II) and the AC threshold pre- (*r* = 0.31; *p* = 0.003, Fig. 3b) and postoperatively (*r* = 0.033; *p* = 0.01, Fig. 3d) was observed. No such association was found for the ZCMEI-21 score and the ABG preoperatively (*p* = 0.66) or postoperatively (*p* = 0.57) nor for the ZCMEI-21 shift and the AC shift (*p* = 0.19, Fig. 3e) or the ABG shift (*p* = 0.62). Further, neither the hearing subscore shift and the AC shift (*p* = 0.20, Fig. 3f), nor the hearing subscore shift and the ABG shift (*p* = 0.67) correlated with each other.

## Discussion

Classification systems for cholesteatoma aim to facilitate the comparison of surgical outcome and the success rate of individual surgical methods [13, 15]. Additionally, they may be helpful for surgical planning and in predicting outcomes [15]. This study aimed to map HRQoL to the cholesteatoma classification ChOLE [15]. With missing significant associations between the ChOLE classifications and HRQoL as assessed by the disease-specific questionnaire ZCMEI-21, this study confirms the clinical observation that the extent of the disease does not necessarily correlate with the subjective complaints of the patient. This circumstance—in particular if preoperative symptoms are mild—impedes the preoperative counseling aiming at recommending surgery, which is necessitated by the sole fact of the presence of a cholesteatoma. We are not aware of any studies systematically investigating the relationship between the cholesteatoma extent and HRQoL. Systematic studies addressing the HRQoL in cholesteatoma surgery are sparse, lack prospective evaluations and/or are mainly studying the influence of surgical techniques on HRQoL [18, 20, 27, 30, 31]. Lucidi et al. as well as Lailach et al. focused on the surgical approach and assessed HRQoL postoperatively using the Chronic Ear Survey (CES) and the Chronic Otitis Media Outcome Test (COMOT-15) [20, 31]. Westerberg et al. investigated the postoperative HRQoL in patients undergoing canal wall up cholesteatoma surgery [30]. Nadol et al. assessed HRQoL in a prospective setting comparing patients with active and inactive COM. The most significant findings include lower changes in HRQoL in patients with inactive COM compared to patients with cholesteatoma [18]. Further, a recurrently draining ear has a major impact on HRQoL, which is in accordance with the findings of one of our previous studies [32]. Additionally, a correlation between the hearing outcome and HRQoL is reported.

The results of this study show that larger cholesteatomas tend to have poorer hearing thresholds preoperatively. This effect can no longer be observed with the postoperative values after hearing restoration and regarding the shifts. It is hypothesized that the results of hearing restoration are comparable independently from the size of the cholesteatoma. This finding is well in accordance to existing studies demonstrating that even after largely extended cholesteatoma, a satisfying hearing restoration can be achieved [33].

Additionally, this study showed that a poorer status of the ossicular chain is associated with poorer pre- and postoperative hearing thresholds confirming earlier investigations on this concern [17]. Further, a trend to a poorer HRQoL associated with a poorer status of the ossicular chain was observed. Studies addressing the outcome of tympanomastoid surgery report that a preserved stapes suprastructure may lead to better hearing outcomes; whereas, large perforations, otorrhea and an absent malleus handle predict a worse audiological performance [7, 12, 34, 35] and have an influence on the type of reconstruction [33]. These findings highlight the importance of the ossicular chain and are supported by the results of this study.

With regard to predicting hearing outcome, three different subgroups (hearing deterioration, improvement and no change) were analyzed. Within these groups, no differences concerning the cholesteatoma stage, extent (Ch) and ossicular status (O) were observed. Therefore, the classification may not be used as a predictive measure of the hearing outcome. Nonetheless, the classification is considered as a valuable additional tool to compare the surgical assessment since the results of this study lead to the assumption that the cholesteatoma stage is not the only factor influencing hearing outcome and the subjective complaints. Only the combination of cholesteatoma staging, hearing outcome and HRQoL may yield a valuable and comprehensive statement regarding all the relevant aspects of the disease and its treatment.

Interestingly, strong associations between HRQoL and hearing thresholds were observed. These results lead to the assumption that hearing has a large impact on the individual HRQoL [1, 36] and may be taken into account when considering primary hearing restoration. Disease-specific symptoms of hearing impaired patients that influence HRQoL include tinnitus, vertigo, hyper-/dysacusis, which have been also reported for otosclerosis [37–39]. It is well known, that restoration of hearing using hearing aids has a positive impact on HRQoL [40, 41]. In addition, there are multiple studies describing an improvement of HRQoL after hearing restoration with active bone conduction or middle ear implants [42, 43]. Nevertheless, these implants do not aim to replace successful hearing restoration in middle ear surgery [44]. A disease-specific evaluation of HRQoL is advantageous to assess additional information that can be used to determine individual complaints and expectations from surgery [18, 19]. Ascertaining the ZCMEI-21 score at different time points may help to measure the patient-reported dimension of the disease and complements audiometry and medical history.

This study has several limitations. First, only few patients with stage I and stage III cholesteatoma were observed, whereas the majority was classified stage II. Yet, this is in accordance with the ChOLE stage distributions found in other studies [15, 17]. Thus, the statistical analyses are to be interpreted with caution concerning stage I and stage III cholesteatomas. Nevertheless, a number of 18 supra-/infralabyrinthine and petrous apex cholesteatomas (Ch4) is reported in this cohort, and neither a significant influence on the preoperative hearing or the hearing outcome nor on the patient’s subjective complaints was observed. Taken together, the present study cohort reflects well the distribution of cholesteatomas and the small incidence of largely extended cholesteatomas in our highly developed countries [10, 45]. Moreover, multiple statistical tests were performed without using a procedure to adjust for multiple testing. Therefore, this study may carry the risk for a type I error. However, we strongly consider our study as an exploratory study and thus accept the risk for a type I error with the benefit of the negligible risk of a type II error, which is crucial for an exploratory, hypotheses generating study, such as the present one. Hypotheses generated by this study may further be investigated by future studies using rigorous adjusting methods for multiple testing. Lastly, the data of this study show wide spread follow-up periods. The postoperative follow-up period is influenced by the speed of healing, granulation tissue and possible complications. Long-term results may depend on other factors such as the dislocation of prostheses, scar tissue or atrophy of the transplant. For this reason, determining the ideal follow-up date is challenging. In addition, some patients are referred for follow-up to private practitioners as soon as the postoperative site is unremarkable and may be lost to follow-up. However, the ZCMEI-21 questionnaire refers to the past 14 days and was only distributed to patients who had finished primary follow-up with uneventful healing and dry microscopic ear findings without any signs of inflammation.

## Conclusion

In conclusion, the clinical applicability of the ChOLE classification was further evaluated, which is easy and comprehensive. The ChOLE classification is not strongly associated with HRQoL but correlates well with the AC PTA. We support the further clinical application of both the ChOLE classification and the ZCMEI-21 in particular under the aim of quality control and generation of comparable data sets in middle ear surgery.
